# The effect of polymerization temperature and reaction time on microwave absorption properties of Co-doped ZnNi ferrite/polyaniline composites†

**DOI:** 10.1039/c8ra05500a

**Published:** 2018-08-17

**Authors:** Yiming lei, Zhengjun Yao, Haiyan Lin, Jintang Zhou, Azhar Ali Haidry, Peijiang liu

**Affiliations:** College of Materials and Technology, Nanjing University of Aeronautics and Astronautics Nanjing 211100 China 121leiyiming@nuaa.edu.cn imzjt@126.com +86 139 5181 8597; Key Laboratory of Material Preparation and Protection for Harsh Environment (Nanjing University of Aeronautics and Astronautics), Ministry of Industry and Information Technology Nanjing 211100 China; Research Institute of Aerospace Special Materials & Technology Beijing 100074 China

## Abstract

This study presents the systematic potential effects of reaction parameters on the synthesis of Co-doped ZnNi ferrite/polyaniline composites prepared *via* novel interfacial polymerization. Through intensive experiments and analysis, optimum reaction conditions including the polymerization temperature and reaction time are proposed so that the performance of the material is significantly improved. The structure, functional groups and morphologies of composites are investigated by X-ray photoelectron spectroscopy (XPS), X-ray diffraction (XRD), Fourier transform-infrared (FT-IR) spectroscopy, scanning electron microscopy (SEM) and high resolution transmission electron microscopy (HRTEM). In addition, the electromagnetic properties and microwave absorption properties of Co-doped ZnNi ferrite/polyaniline composites are examined by a vibrating sample magnetometer (VSM), Quantum Design (MPMS-VSM and MPMS-XL), the superconducting quantum interference device (SQUID) magnetometer and vector network analysis. Based on these analyses, it is found that by tuning the reaction conditions, *i.e.*, polymerization temperature and reaction time, microwave absorption capabilities in terms of the maximum reflection loss (*R*_L_) value and absorber thickness can be readily optimized. The results show that the composite with an optimized polymerization condition of 20 °C for 12 h displays remarkable microwave absorption properties with maximum reflectivity of −54.3 dB, and the effective bandwidth (*R*_L_ < −10 dB) is about 6.02 GHz at a thickness of 6.8 mm. Furthermore, the discussion shows that the promising microwave absorption may be due to the uniform urchin-like structure of the composites.

## Abstract 2

This study presents the systematic potential effects of reaction parameters on the synthesis of Co-doped ZnNi ferrite/polyaniline composites prepared *via* novel interfacial polymerization.

## Graphical abstract

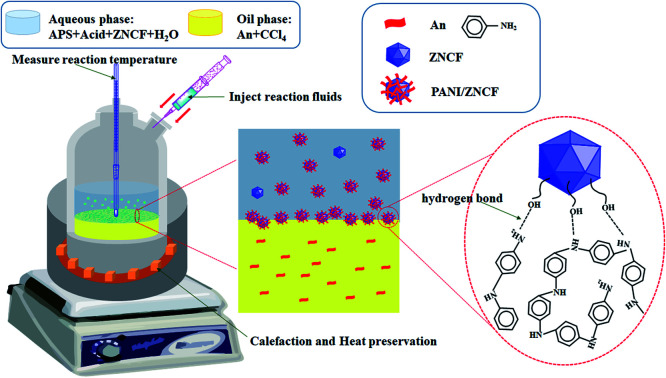

## Introduction

In the modern world, with a growing number of wireless technologies in our daily life, scientists are becoming increasingly concerned about the consequences of electromagnetic (EM) pollution that has caused very serious health and environmental problems such as Lyme disease, chronic fatigue syndrome, and damage to other various human physiological systems.^1,2^ Therefore, in recent years, high-performance EM wave absorbing materials having low thickness, wide bands, light weight, and strong absorption properties have been intensively studied.^3^ The EM wave can be absorbed by some organic/inorganic materials (such as graphene, carbonyl iron powders, *etc.*) through dielectric (and magnetic) losses, and the material can exhibit the best EM wave absorption while satisfying the impedance match.^4^

Among the various EM wave absorbing materials, spinel ferrites have been widely explored due to their excellent magnetic spectrum and thin absorbing layers.^5^ Spinel ferrites belong to the cubic crystal system with high symmetry and small anisotropy of magnetic crystals. They are typically represented as MeFe_2_O_4_ (Me = Zn^2+^, Ni^2+^, Co^2+^, Mg^2+^, *etc.*).^5^ The EM wave absorption properties of spinel ferrites are not only related to their elemental composition, but are also affected by their microstructure, particle size, and other factors.^6^ For example, Xie *et al.*^7^ synthesized (Ni_0.407_Co_0.207_Zn_0.386_)Fe_2_O_4_ ferrite that exhibited an effective absorption frequency band (reflection loss below −10 dB) from 8.64 to 11.2 GHz. In another report, Sozeri *et al.*^8^ studied Mn–Co-substituted Ni–Zn ferrite nanoparticles with a simple format Ni_*x*_Zn_0.8−*x*_Mn_0.1_Co_0.1_Fe_2_O_4_ (0 ≤ *x* ≤ 0.8), and the results showed that Ni_0.6_Zn_0.2_Mn_0.1_Co_0.1_Fe_2_O_4_ has the best EM wave absorption properties with the maximum reflection of −25 dB at frequency 10 GHz. In that study, Ni–Zn–Co ferrite was used due to its high magnetic loss, and EM wave was absorbed by eddy current loss, hysteresis loss and natural resonance. Compared with Ni–Zn ferrite, the doped Co^2+^ ferrite could increase the saturation magnetization strength, coercivity, and dielectric constant.^9^ However, issues such as narrow EM wave absorption bands and high density limit their application potential in electronic communication and radar stealth industries. One of the effective ways to solve such problems is to combine conductive polymer materials with ferrites as they have low density, a strong designed structure, easy processed form, and unique electrical properties.

Polyaniline (PANI) is a typical dielectric material among conducting polymers known for its excellent environmental stability, facile synthesis condition, and controlled electrical conductivity.^10^ The conductive PANI/ferrite composites have increasingly attracted more attention because of the synergistic effect between PANI and ferrite.^11,12^ For example, Ting *et al.*^13^ synthesized an NiZn ferrite material coated with different ratios of PANI, which showed that a wider EM wave absorption band could be achieved by adding various ratios of PANI in the frequency range of 2–40 GHz. Wang *et al.*^14^ fabricated Ni_0.5_Zn_0.5_Fe_2_O_4_/PANI nanocomposites through the hydrothermal method, and the results exhibited an effective absorption bandwidth at 5 GHz. Yang *et al.*^15^ prepared a BaFe_12_O_19_/Y_3_Fe_5_O_12_ composite coated with PANI and found the maximum reflection loss to be −40.8 dB at 9.9 GHz.

It is well-known that pure PANI is almost an insulator, but appropriate acid doping can improve its conductivity. In addition, interfacial polymerization has many advantages as compared with traditional *in situ* polymerization. For instance, the polymerization occurs at the interface of two dissolvable solvents because aniline monomers can only contact the oxidant on the interface and subsequently, the reaction occurs. Upon formation of hydrophilic PANI nanofibers, they can quickly leave the interface and diffuse to the aqueous phase, thus avoiding the secondary growth of nanometer fibers. In this context, the current report proposed facile synthesis of Zn_0.4_Ni_0.4_Co_0.2_Fe_2_O_4_ (ZNCF) particles *via* the cost-effective sol–gel method. Thereafter, ZNCF/PANI nanocomposites were prepared by the interfacial polymerization procedure. In addition to this, to the best of our knowledge, this is the first time that the effects of polymerization temperature and reaction time on EM wave absorption properties are explored. Furthermore, the mechanism of enhanced EM absorption behavior is discussed in detail on the basis of structural, morphological, and electromagnetic properties.

## Materials and methods

### Materials

Aniline, commercial ferric nitrate [Fe(NO_3_)_3_·9H_2_O], NaNO_3_ and KMnO_4_ were purchased from Aladdin Chemical Reagent, China. Zinc nitrate [Zn(NO_3_)_2_·6H_2_O] (AR, 99%), cobalt nitrate [Co(NO_3_)_2_·6H_2_O] (AR, 99%), nickel nitrate [Ni(NO_3_)_2_·6H_2_O] (AR, 98%), carbon tetrachloride [CCl_4_] and citric acid (C_6_H_8_O_7_) with purity > 99.5% were supplied by Adamas Industrial Corporation Chemical Co. Ltd, China. Ammonium persulfate (NH_4_)_2_ S_2_O_8_ with purity > 98.5% was purchased from Nanjing Chemical Reagent Co. Ltd, China. All the chemicals were used without further processing except aniline, which was purified by vacuum distillation before use.

### Synthesis of ZNCF

Micron-sized ZNCF particles were fabricated by the sol–gel method. The typical ferrite has a relatively superior performance with the molar ratio of 0.4 : 0.4 : 0.2 : 2, corresponding to the ions Zn^2+^ : Ni^2+^ : Co^2+^ : Fe^3+^, which constitute Zn_0.4_Ni_0.4_Co_0.2_Fe_2_O_4_.^16^ A typical ZNCF preparation process is as follows: first appropriate stoichiometric ratios of all nitrates were dissolved in 120 mL deionized water with constant stirring for 5 min, followed by quantitative citric acid accession under continuous stirring for another 10 min. Then, NH_3_·H_2_O was added to the above solution with continuous stirring to adjust the pH value to 7. The suspension was then poured into a dry glass-beaker and heated in an oil-bath at 80 °C for 7 h to form a gel-state mixture. Subsequently, two heat treatments were used to relax the mixture: it was dried in an oven at 130 °C for 11 h and heated at 210 °C for 2 h. Finally, the material was annealed at 1090 °C for 2 h and cooled in the air. ZNCF was used for further preparation.

### Synthesis of PANI/ZNCF composites

**Fig. 1:**
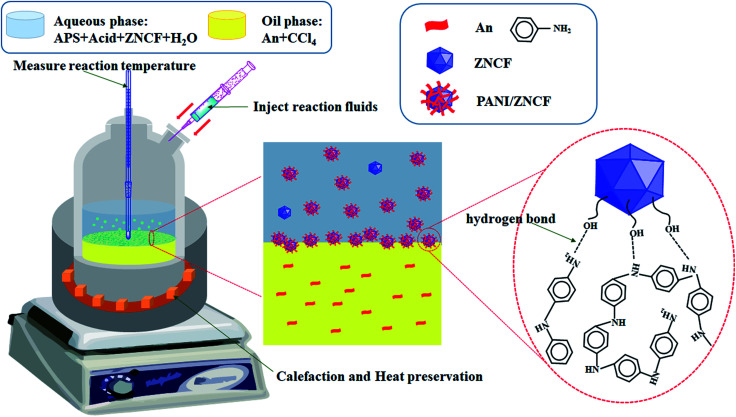
The schematic shows synthesis steps and combination of chemical bonds of PANI/ZNCF composites.

In a typical procedure, 0.2 g aniline (An) was dissolved in 50 mL carbon tetrachloride (CCl_4_) with stirring for 20 min to form a solution, which was named solution A. Then, ratable (NH_4_)_2_S_2_O_8_ (APS) and ZNCF (molar ratio of the APS/aniline was 1 : 1) were added to 50 mL distilled water, which was named as solution B, along with continuous stirring for 10 min. Thereafter, appropriate content of HCl (1 mol L^−1^) was dissolved in the above solution B with stirring for 20 min at room temperature. Polymerization started after the dropwise addition of solution B onto the surface of solution A, resulting in the formation of layered solutions under controlled reaction times and temperatures, which are listed in Table 1. Finally, the suspension was filtered and cleaned with distilled water until the filter liquor became clear, followed by drying in a drying cabinet at 60 °C for 24 h. The schematic diagram of the preparation of PANI/ZNCF composites is shown in Fig. 1.

**Table 1:** The preparation of PANI/ZNCF composites under different conditions

Sample	Reaction time	Polymerization temperature
T-1	12 h	0 °C
T-2	12 h	20 °C
T-3	12 h	40 °C
D-1	4 h	20 °C
D-2	8 h	20 °C
D-4	16 h	20 °C
D-5	20 h	20 °C
D-6	24 h	20 °C

### Characterization

Heteroatom functional groups and element identification were characterized by X-ray photoelectron spectroscopy (XPS, Thermo ESCALAB 250XI). X-ray diffraction (XRD) of prepared samples was performed by using a Bruker D8 X-ray diffractometer with Cu Kα radiation (*λ* = 0.15406 nm) in the 2*θ* range (2*θ* = 10–80°) followed with a scanning rate of 2° min^−1^. The chemical bonds and functional groups of samples were analyzed by Fourier transform-infrared (FT-IR) spectra in the range of 2000–400 cm^−1^ using an infrared spectrophotometer (NICOLET, model NEXUS 870) by mixing samples with proper KBr powders to make a slice. The surface morphology of ZNCF and its composites were observed by field effect scanning electron microscopy (Hitachi S-4800N FE-SEM), where the samples were prepared by dispersing the powders in distilled water under stirring for 15 min and then adding the suspension on the surface of a silicon wafer (height: 5 mm, width: 5 mm). The morphology and details of composites were observed by a high resolution transmission electron microscope (HRTEM, JEM 2100F), and the samples were prepared by ultrasonic suspension in ethanol. The hysteresis loops of samples were investigated by a vibrating sample magnetometer (VSM, Lake Shore) with a magnetic field ±10 kOe, where the samples were prepared by covering 0.2 g powders with wipe papers, sealing with paraffin solution and trimming the samples into cubes (height: 5 mm, width: 3 mm, and thickness: 1 mm). Magnetic properties were measured by the Quantum Design (MPMS-VSM and MPMS-XL) the superconducting quantum interference device (SQUID) magnetometer in an applied field (100 Oe). The complex permittivity and permeability were measured using VNA (Agilent PNA N5224A) in the frequency range of 2–18 GHz. The test circular ring was composed of wax and samples with a mass ratio of 7 : 3 following an artificial hot pressing progress in the mold, and it was cooled at room temperature. Finally, the test ring with inner diameter 3.01 mm, outer diameter 7.02 mm and thickness 2 mm was obtained.

## Results and discussion

**Fig. 2:**
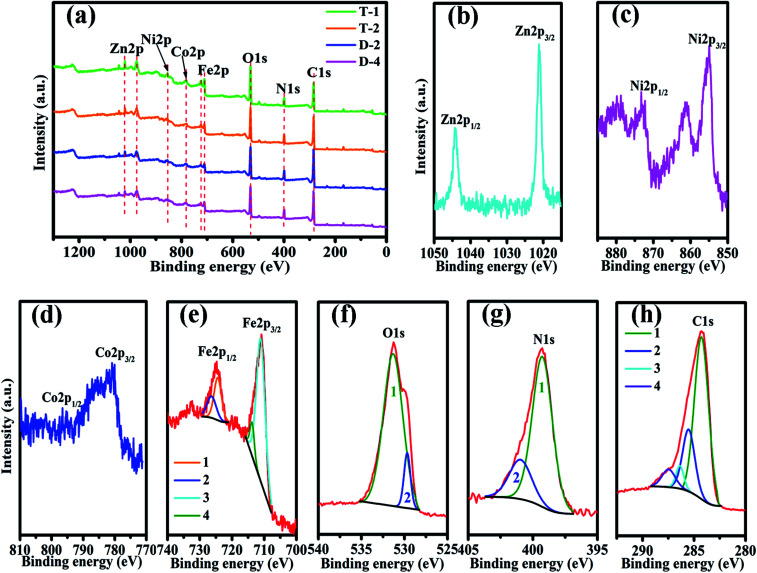
The XPS spectra of (a) sample T-1, T-2, D-2 and D-4, (b) Zn 2p, (c) Ni 2p, (d) Co 2p, (e) Fe 2p, (f) O 1s, (g) N 1s, and (h) C 1s of sample T-2.

Fig. 2 shows the elemental components of PANI/ZNCF composites measured by XPS. In Fig. 2a, the XPS spectrum of composites shows sharp peaks at 1021.2, 855.1, 780.1, 713.9, 531.2, 398.2, and 284.2, which correspond to the characteristic peaks of Zn 2p, Ni 2p, Co 2p, Fe 2p, O 1s, N 1s, and C 1s. These diffraction peaks indicate the existence of Zn, Ni, Co, Fe, O, N, and C in the composites.^17^ After polymerization, all composites exhibit both ZNCF and PANI diffraction peaks, and the positions of these peaks have negligible shifts. No other clear characteristic peaks can be found in the XPS spectrum. Fig. 2b indicates the presence of two peaks at 1044.4 eV and 1021.2 eV, corresponding to the binding energies of Zn 2p_1/2_ and Zn 2p_3/2_. Ni 2p_1/2_ and Ni 2p_3/2_ peaks at 873.1 eV and 854.9 eV, respectively, indicate the presence of Ni^2+^ in the system (Fig. 2c). In the Co spectra (Fig. 2d), the binding energies of 796.5 eV and 780.6 eV are assigned to Co 2p_1/2_ and Co 2p_3/2_, respectively.^17^ The presence of Fe in the samples is confirmed by the peaks at 724.5 eV and 711.3 eV, which are assigned to Fe 2p_1/2_ and Fe 2p_3/2_, respectively, and related to Fe^3+^ ions in tetrahedral sites (plots 1 and 3 in Fig. 2e).^20^ Moreover, curves 2 and 4 show binding energies of 726.7 eV and 714.2 eV, which are assigned to Fe 2p_1/2_ and Fe 2p_3/2_ and related to Fe^3+^ ions in octahedral sites.^7,19,20^ The O 1s spectrum (Fig. 2f) can be resolved into two peaks centered at 531.6 eV and 529.8 eV, which are assigned to the surface hydroxyl and oxygen groups, respectively, in the composite.^21,22^ The analysis of the N 1s spectrum (Fig. 2g) reveals two binding energies: 400.1 eV (curve 2) and 399.3 eV (curve 1). The binding energy in curve 2 is assigned to the benzene–diamine units, whereas that in curve 1 is due to the quinine–diimine units.^23^ The C 1s spectrum (see Fig. 2h) can be resolved into four peaks at different binding energies. The curve 1 with a peak at 284.3 eV corresponds to C–C or C–H bonds. The curve 2 shows a peak at 285.6 eV, which is assigned to C–N or C=N bonds, and the third one (curve 3) at 286.5 is due to the C–O bond; the forth one (curve 4) at 287.5 corresponds to C=N^+^ or C=O bonds.^18,23^

**Fig. 3:**
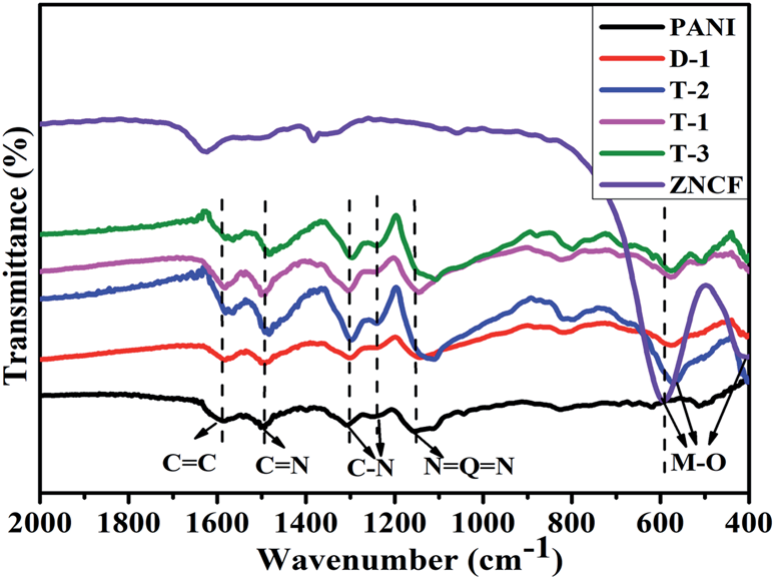
The FTIR spectra for PANI, NZCF, and samples D-1, T-1, T-2, and T-3.

The XRD patterns of the obtained PANI/ZNCF composites are shown in Fig. S1.† The analysis of XRD patterns indicates the presence of typical spinel cubic-structure ferrites in the composites. The FT-IR spectrum of pure PANI, ZNCF and their composites is shown in Fig. 3. The spectrum of ZNCF indicates two characteristic peaks, whereas the peaks at 590 and 410 cm^−1^ can be assigned to the coupling between metal and oxygen (M–O) stretching modes of the spinel structure.^13,17^ The peaks at 1587 and 1496 cm^−1^ indicate the C=C bond of the benzenoid ring and C=N bond of the quinoid ring, respectively.^24^ The characteristic peaks at 1306 and 1233 cm^−1^ indicate the C–N stretching vibration of benzenoid ring.^24,25^ The distinct peak at 1150 cm^−1^ is described as“electronic-like band”,^26^ which is assigned to the N = Q = N mode (Q on behalf of the quinonic-type rings). The PANI/ZNCF composites exhibit both PANI and ZNCF characteristic peaks, whereas there is a clear shift at 564 cm^−1^ because the PANI chains are tightly covered with ZNCF.^27^ This result is consistent with the XRD results, indicating that the PANI/ZNCF composites are successfully synthesized.

**Fig. 4:**
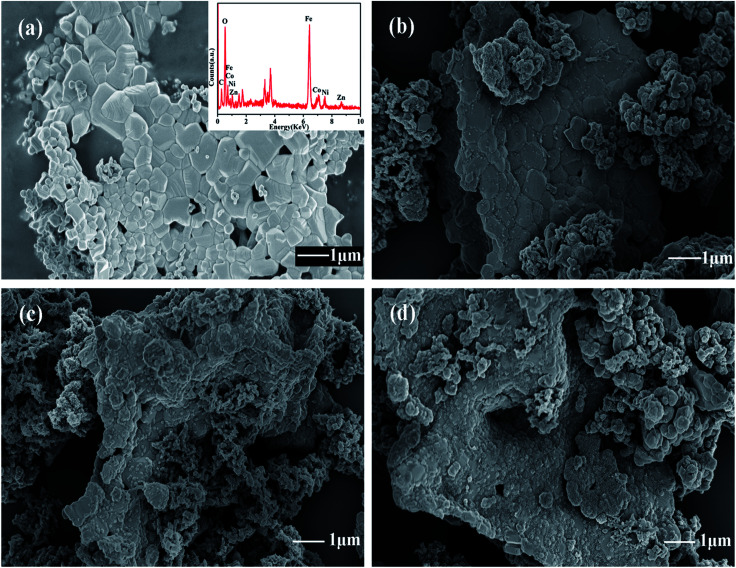
The SEM micrographs of (a) ZNCF (the inset shows its corresponding EDS spectrum), (b) T-1 (c) T-2 and (d) T-3 samples.

To investigate the effect of temperature on polymerization of PANI/ZNCF composites, SEM results of ZNCF and composites synthesized at 0 °C, 20 °C and 40 °C for 12 h are obtained. Fig. 4a shows the SEM micrograph and EDS spectrum of ZNCF, where ZNCF particles exhibit a cubic-like structure with a smooth surface, and they are tightly bound because magnetic particles attract each other.^11^ The diameter of the ZNCF granules ranges from 400 nm to 800 nm. The EDS spectrum indicates that Zn, Ni, Co, and Fe exhibit 2 characteristic peaks, which are consistent with XRD results. As seen in Fig. 4b, PANI molecular chains agglomerate and disperse unevenly on the surface of ZNCF particles, due to which some ZNCF particles are exposed to air, which causes negative effect on the absorption of electromagnetic waves.^21,22,28^Fig. 4c and d exhibit similar urchin-like structures that are ZNCF granules covered with uniformly distributed PANI chains and a number of salient forms on the surface of particles. However, some accumulation of PANI chains is observed for both samples, and sample T-3 agglomerates seriously because the molecular thermodynamic movement is promoted with the increase in temperature. Compared with the above samples, the sample dried at 20 °C (T-2) has the most homogeneous stable structure, which has a significant effect on its performance. To confirm the optimum reaction conditions, a series of contrast experiments under different reaction times at 20 °C are carried out.

**Fig. 5:**
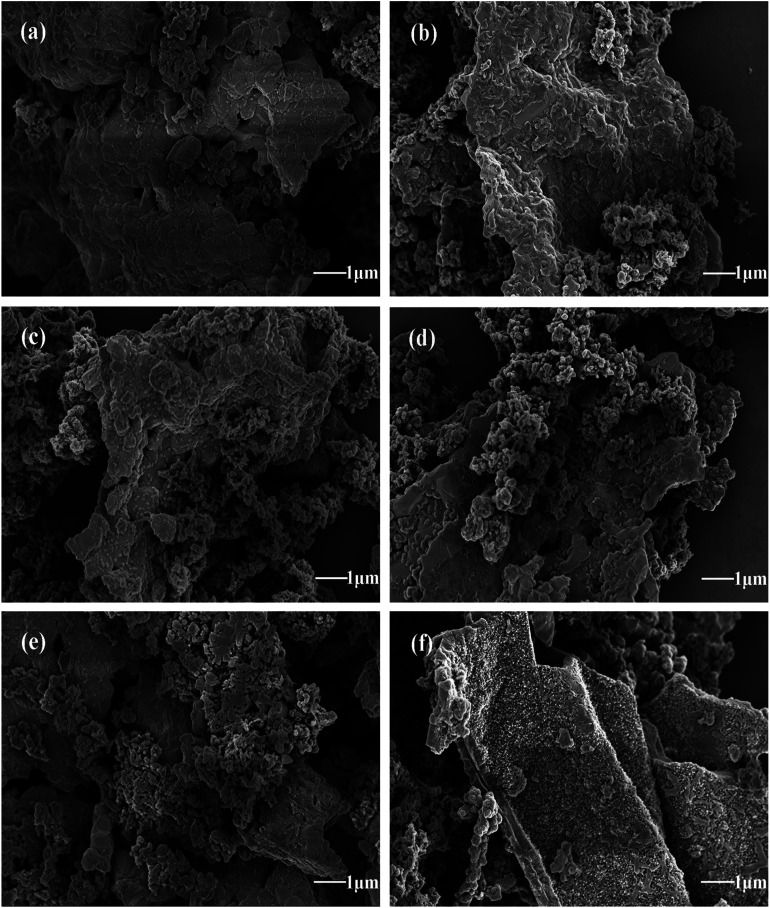
The SEM images of (a)–(f) indicate composites synthesized under different reaction times (D-1, D-2, T-2, D-4, D-5 and D-6, respectively).

Morphological characteristics of formed PANI/ZNCF composites under different reaction times (4 h, 8 h, 12 h, 16 h, 20 h, and 24 h) can be observed in Fig. 5. It can be seen that PANI/ZNCF composites exhibit a rough surface, which demonstrates that the composites are compounded successfully by interfacial polymerization. With increasing reaction time (Fig. 5a–c), the ZNCF particles are wrapped by more PANI chains on the surface. As shown for sample D-1 (Fig. 5a), there are few PANI chains on ZNCF; on the other hand, the polarization between PANI chains induces strong agglomeration, which leads to the smooth surface of ZNCF.^13,29^ Samples D-2 and T-2 (Fig. 5b and c) exhibit similar urchin-like structures, but the PANI chains of T-2 disperse better. Furthermore, after increasing the reaction time for D-4, D-5, and D-6 (Fig. 5d–f), the agglomeration increases to a great extent. Low-molecular-weight PANI chains polymerize into larger spheres (the diameter of the PANI particles is increased from 300 nm to 700 nm), which results in ZNCF particle formation. In this study, the PANI/ZNCF composite prepared with the reaction conditions of 12 h and polymerization temperature of 20 °C has a uniformly distributed urchin-like structure, which can be helpful to improve EM wave absorption properties.

**Fig. 6:**
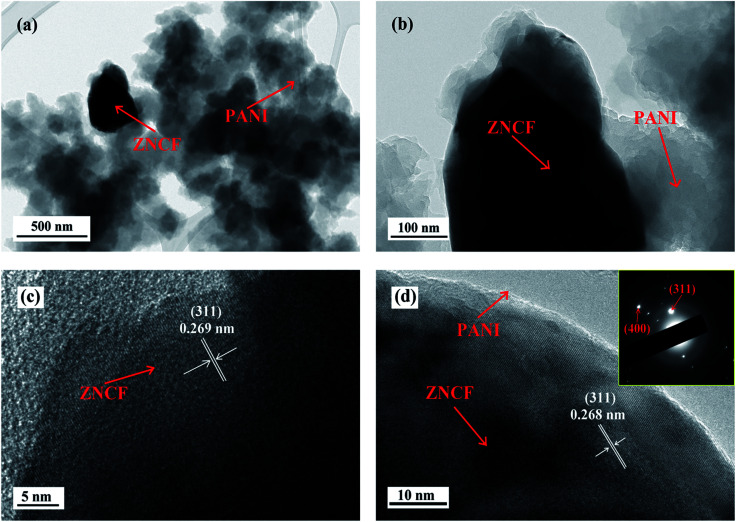
The TEM images of (a and b) sample T-2 and (c and d) HRTEM image of sample T-2 (the inset shows the SAED pattern of sample T-2).

The structure and morphology of sample T-2 are investigated by TEM, and the results are shown in Fig. 6. As shown in Fig. 6a and b, it is clear that some fibrous PANI chains cover ZNCF particles, indicating the formation of an urchin-like structure. To further confirm the crystalline structure of ZNCF, HRTEM image of PANI/ZNCF composites is presented in Fig. 6c and d. Clear lattice fringes can be observed, and the lattice fringe spacing of ZNCF particles is approximately 0.268 nm, corresponding to the (311) plane of ZNCF (Fig. S1†).^21^ The inset shows the SAED pattern of sample T-2, and the (311) and (400) planes can be observed.

**Fig. 7:**
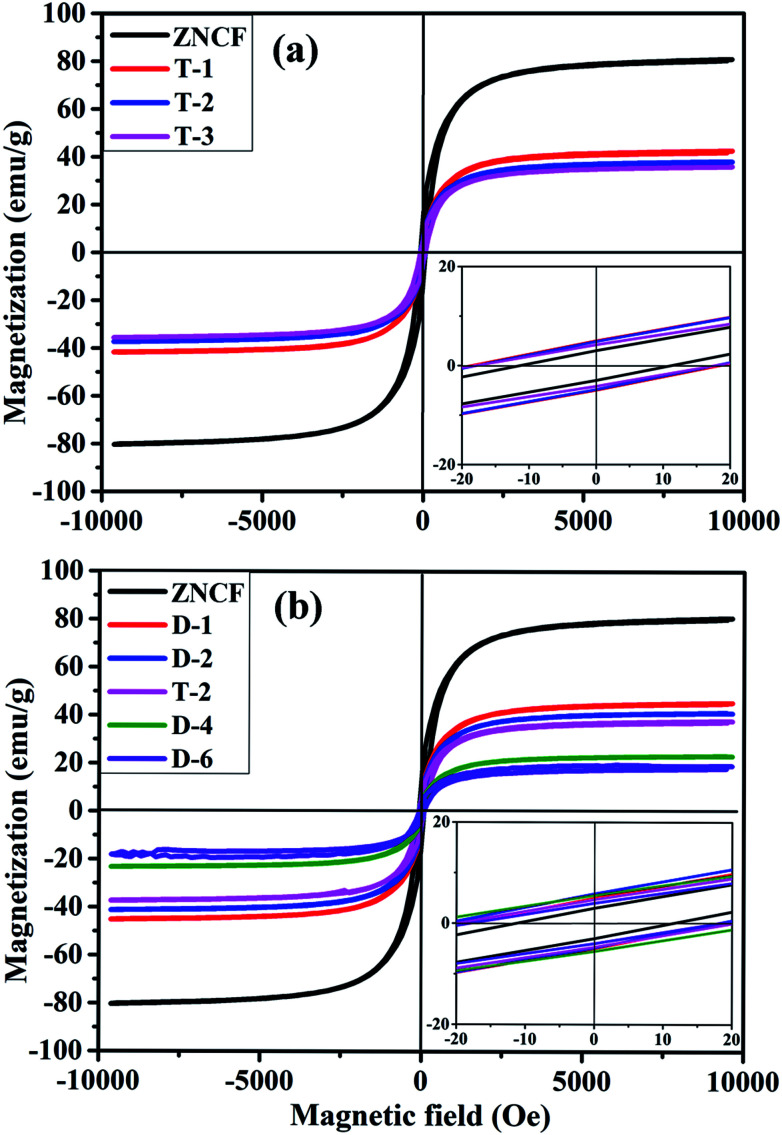
Magnetic hysteresis loops of (a) ZNCF and PANI/ZNCF composites under different polymerization temperatures and (b) ZNCF and PANI/ZNCF composites under different reaction times (the inset is the enlarged view of the hysteresis loops at low applied fields).

Hysteresis loops of PANI/ZNCF composites under different reaction temperatures are shown in Fig. 7a. The samples ZNCF, T-1, T-2, and T-3 exhibit ferromagnetic behaviors with saturation magnetization values of 81.1, 42.1, 38.1, and 35.5 emu g^−1^, respectively. The coercivity of all samples is negligible, which can be used in soft magnetic field.^21,27^ The saturation magnetization of ZNCF is much higher than that of composites owing to magnetism that is reduced by additional nonmagnetic PANI.^30^ Furthermore, Fig. 7b shows the magnetic properties of samples ZNCF, D-1, D-2, T-2, D-4, and D-6 with saturation magnetization values of 81.1, 45.6, 41.2, 38.1, 23.3, and 18.1 emu g^−1^, respectively. A possible explanation is that ZNCF is covered with increasing nonmagnetic PANI chains, leading to decreased magnetic properties. Besides, there may be a critical value of additional reaction time for which saturation magnetization is almost unchanged.^31^ The coercivity values of these samples are low (∼10–20 Oe).

**Fig. 8:**
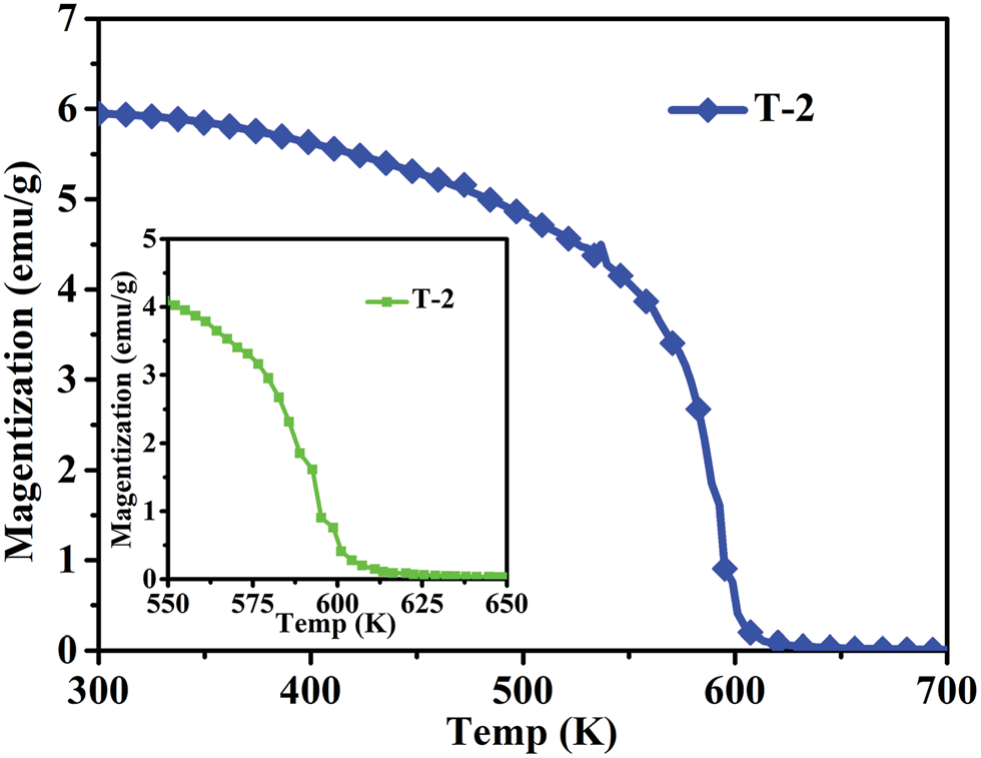
Temperature dependence of magnetization in the range of 300–700 K of sample T-2 (the inset is the temperature dependence of magnetization in the range 550–650 K of T-2).

Fig. 8 shows the magnetization of sample T-2 *versus* temperatures in an applied field (100 Oe). The result shows a clear transformation phase from magnetic order to disorder from 300 K to 700 K. The inset shows that the composite has a Curie temperature of about 593 K. The sample T-2 shows relatively stable magnetization at low temperatures (300–400 K). A similar result has been reported by Li *et al.*^32^ for Ni_0.4_Zn_0.5_Co_0.1_Fe_2_O_4_ ferrite prepared by the sol–gel method. However, in this study, the Curie temperature of the composite is higher than that reported, which may be due to different ratios of ferrite.

**Fig. 9:**
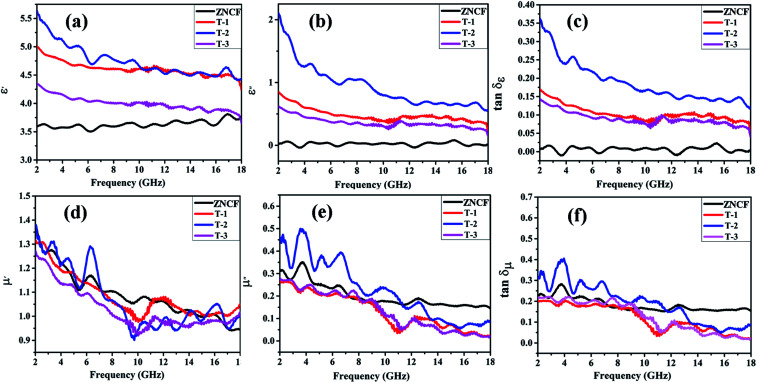
Frequency dependence of the real part (a) and imaginary part (b) of complex permittivity, dielectric loss tangent (c), the real part (d), imaginary part (e) of the relative complex permeability and magnetic loss tangent (f) of ZNCF and PANI/ZNCF composites under different polymerization temperatures.

Fig. 9a and b show the real (*ε*′) and imaginary part (*ε*′′) values of complex permittivity under different polymerization temperature conditions in the frequency range of 2–18 GHz. It is well-known that *ε*′ represents the storage and *ε*′′ represents the loss ability of electric energy. The *ε*′ value of ZNCF is almost unchanged, and the *ε*′′ value is fairly low (approaching almost zero); this indicates that ZNCF is not a dielectric loss-absorbing material.^33^ Compared with ZNCF, PANI/ZNCF composites exhibit relatively better dielectric properties due to the doped PANI chains. A number of π–π conjugated bonds found in PANI are favorable to decrease collision of electrons.^12^ According to the free electron theory, more conductive PANI chains increase the complex permittivity value.^34,35^ As shown in Fig. 9a and b, sample T-2 exhibits higher *ε*′ and *ε*′′ values compared with T-1 and T-3, confirming that dielectric properties can be influenced by reaction temperature and there exists an optimal temperature (20 °C). According to the authors, the urchin-like coated structures of PANI/ZNCF composite and grafted PANI chains offer extra contact surfaces and junctions as compared with other composites.^36^ The dissipation factors varying with frequency can be seen as dielectric loss tangent (tan *δ*_ε_ = *ε*′′/*ε*′) (Fig. 9c). The T-2 composite has the maximum tan *δ*_ε_ value. Meanwhile, the values of tan *δ*_ε_ for three PANI/ZNCF composites are greater than that for pure ZNCF.

Fig. 9d and e exhibit the real part (*μ*′) and imaginary part (*μ*′′) values of complex relative permeability under different polymerization temperature conditions (frequency ranges from 2 to 18 GHz). As shown in Fig. 7d, the *μ*′ values of T-1 and T-3 are slightly lower than that of ZNCF, whereas T-2 exhibits higher *μ*′ value as compared with ZNCF in frequency range of 2–7 GHz. Fig. 9e demonstrates decreased trends of *μ*′′ values with the increase in frequency. Sample T-2 exhibits higher *μ*′′, which represents the best magnetic loss. However, the *μ*′′ values of T-1 and T-3 are lower than that of ZNCF owing to excessive or minimal non-magnetic PANI chains that reduce the magnetism of the composites. The tan *δ*_μ_ value is calculated based on the *μ*′ and *μ*′′ values (tan *δ*_μ_ = *μ*′′/*μ*′) (Fig. 9f); as seen in this figure, changing the polymerization temperature has clear effects on the magnetic loss of PANI/ZNCF composites. The value of tan *δ*_μ_ for sample T-2 is greater than that of ZNCF in the range 2–12 GHz, which ascribes to the charge transfer between the ZNCF surface and PANI.^37^ Sample T-2 exhibits best tan *δ*_μ_ as compared with other composites. Besides, the comparison of all the samples indicates that sample T-2 exhibits similar tan *δ*_μ_ and tan *δ*_ε_ values, which can increase the degree of impedance matching and promote the EM wave absorption properties of materials.

**Fig. 10:**
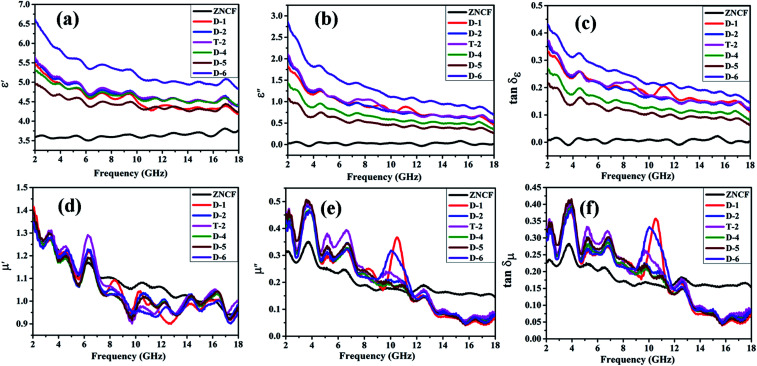
Frequency dependence of the real part (a) and imaginary part (b) of the complex permittivity, dielectric loss tangent (c), the real part (d), imaginary part (e) of the relative complex permeability and magnetic loss tangent (f) of ZNCF and PANI/ZNCF composites under different reaction times.

Fig. 10 shows complex permittivity and complex permeability of ZNCF and its composites under different reaction times in the frequency range of 2–18 GHz. As shown in Fig. 10a and b, sample D-6 has a relatively outstanding *ε*′ value, which decreases from 6.7 to 4.8, and the *ε*′′ value decreases from 2.8 to 0.7 in the frequency range from 2 to 18 GHz. It is reasonable to show that the different dielectric behaviors of this sample are related to its special morphology and structure. Fig. 10c shows the dielectric loss (tan *δ*_ε_) of composites. All composites show similar tan *δ*_ε_ values, exhibiting that the change in reaction time has negligible effects on dielectric loss. Sample D-6 has a slightly higher value of tan *δ*_ε_ compared with others in low frequency, which may contribute to microwave absorption. Fig. 10d and e show *μ*′ and *μ*′′ of PANI/ZNCF composites. It can be seen that both values of *μ*′ and *μ*′′ for all samples exhibit decreased tendencies with increased frequencies. For ZNCF particles, *μ*′ and *μ*′′ retain the relatively little decrease trends as compared with that for PANI/ZNCF composites in the frequency range of 2–18 GHz, and *μ*′ of ZNCF is higher than those of other samples in the range of 7–14 GHz. This specific phenomenon is due to the addition of PANI, reducing the ability of magnetic energy.^38^*μ*′′ generally represents the loss ability of the magnetic material. *μ*′′ of ZNCF is lower than that of the composites in the frequency range of 2–11.5 GHz, but it is higher in the range of 11.5–18 GHz. Nearly all PANI/ZNCF composites exhibit similar decreased tendencies of *μ*′ and *μ*′′ except samples D-1 and D-6, which show prominent peaks in the range of 10–11 GHz. Fig. 10f shows tan *δ*_μ_ of composites. It can be seen that changing the reaction time has negligible effects on magnetic loss for PANI/ZNCF composites. Sample D-1 and D-6 have narrow resonance peaks in the range of 10–12 GHz. Samples D-2 and T-2 exhibit better electromagnetic loss tangent values compared with other composites, which can promote the absorption of EM wave.

The complex relative permeability and magnetic loss tangent (tan *δ*_μ_) exhibit clear decrease in the frequency range of 11–18 GHz, which may be due to exchange resonance, dimensional resonance, and eddy current resonance.^19,39^ For spinel ferrites such as ZNCF, exchange resonance has negligible contribution to the magnetic loss in the high frequency range (11–18 GHz).^16,18^ Moreover, the dimensional resonance can be explained as follows:^19,38^

1 *C* = *λf*

**Fig. 11:**
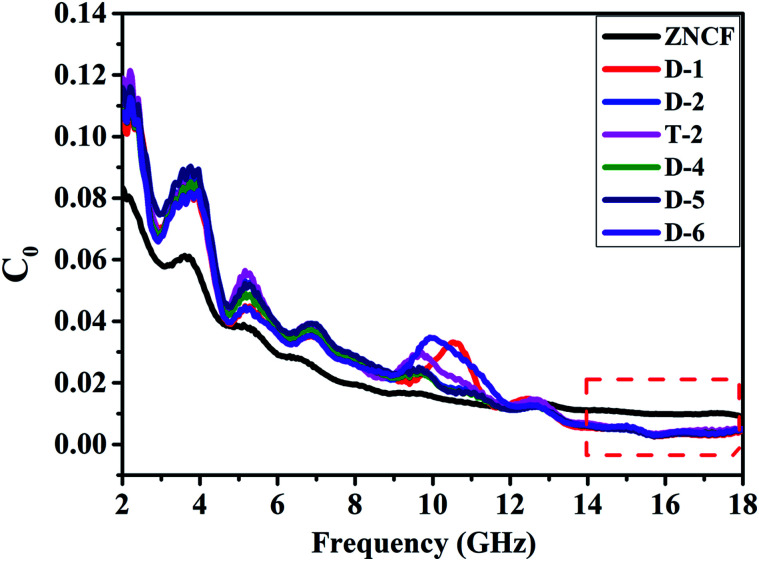
The eddy current data of pure ZNCF and PANI/ZNCF composites with different polymerization temperatures and reaction times.

2 *D* = *nλ*/2 (*n* = 1, 2, 3,…)

here, *d* is the thickness of sample, and *λ* is the wavelength of the electromagnetic wave entering the samples. From eqn (1) and (2), it can be found that the wavelength decreases with the increase in frequency. If the physics origin of the peaks arises from the dimensional resonances, the calculated *d* should be larger than 3 mm, which is much larger than the real sample thickness of about 2 mm. Hence, the dimensional resonance can be excluded.^19^ If the magnetic losses of composites are caused by eddy current resonance, it can be expressed by the equation:^16,19^3
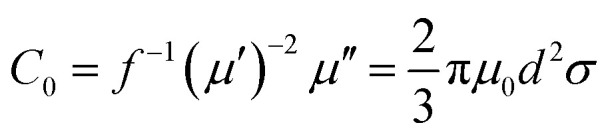
here, *μ*_0_ is the permeability of vacuum, *σ* is the conductivity and *C*_0_ is the eddy current coefficient. The change in *C*_0_ represents that the magnetic loss is not induced by the eddy current loss.^18,19,30^ As can been seen in Fig. 11, the decrease in *C*_0_ at 2–14 GHz indicates that the magnetic loss in this region is not caused by eddy current resonance. When the frequency is higher than 14 GHz, *C*_0_ tends to be stable, proving that the magnetic loss in this region is mainly caused by eddy current resonance.

The microwave absorption properties of as-synthesized PANI, ZNCF and PANI/ZNCF composites can be defined as the reflection loss (*R*_L_), which can be simulated by the complex permittivity and permeability at set thickness according to the transmission theory, as shown in the following equations:^40,41^

4 *R*_L_ (dB) = 20 log|*Z*_in_ − 1/*Z*_in_ + 1|

here, the input impedance *Z*_in_ of the absorber is given by the following equation:5
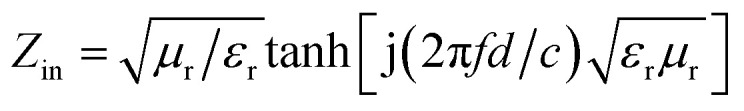
here, the velocity of EM waves in free space is *c*, *d* is the thickness of absorbent layer and *f* is the microwave frequency.

**Fig. 12:**
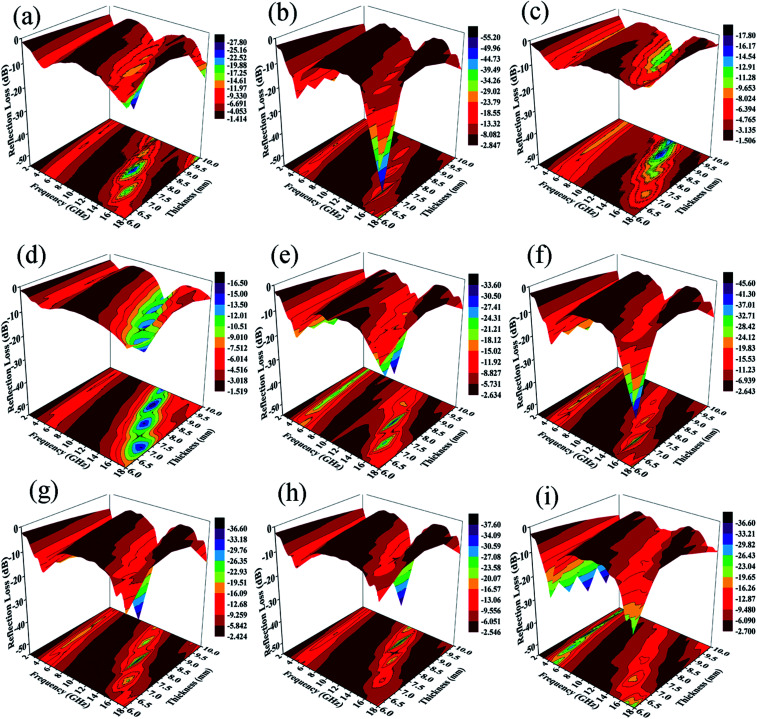
3D representations of reflection loss of ZNCF (d) and samples of PANI/ZNCF composites T-1 (a); T-2 (b); T-3 (c); D-1 (e); D-2 (f); D-4 (g); D-5 (h); and D-6 (i) in the frequency range of 2–18 GHz.

The calculated *R*_L_ values of composites in the frequency range of 2–18 GHz with varied absorber thicknesses of 1–10 mm under different preparation temperatures with 12 h reaction time are shown in Fig. 12a–d. Usually, when *R*_L_ < −10 dB, it results in efficient microwave absorption, which can be used in actual applications. The absorption peak of ZNCF only reaches −17.5 dB with a thickness of 8.9 mm, and the effective bandwidth (*R*_L_ < −10 dB) is about 2.67 GHz. Compared with ZNCF, PANI/ZNCF composites have better microwave absorption properties and especially, the composite prepared at temperature 20 °C is the best. The sample T-2 has an excellent absorption peak, which reaches −54.3 dB with a thickness of 6.8 mm, and the effective bandwidth is about 6.02 GHz. The maximum peak of the T-1 composite reaches −26.3 dB with a thickness of 7.7 mm, and the peak for the T-3 composite reaches −17.2 dB with a thickness of 8.9 mm. Clearly, the urchin-like structure of T-2 has an active effect on the EM wave absorption property.

Fig. 12(e–i) show the EM wave absorption properties of PANI/ZNCF composites with different reaction conditions in terms of preparation time. It is clear that samples D-1, D-2 and T-2 exhibit better microwave absorption properties, and they display decreased trends with the increase in reaction time. The effective bandwidth of sample D-1 is 6.11 GHz, and the maximum *R*_L_ reaches −32.2 dB with a thickness of 7.7 mm. The optimal absorption peak of the D-2 composite can reach −44.1 dB at 15.1 GHz with a thickness of 6.8 mm, and the effective bandwidth can reach 5.71 GHz. Sample T-2 has the best EM wave absorption properties compared to others. From Fig. 12, we can see that the maximum *R*_L_ shifts to the low frequency region with increasing thickness of the sample. Usually, the thicker the sample, the higher the *R*_L_ value for all frequencies. Thus, it is necessary to find a balance between thickness and microwave absorption properties.^42^

**Fig. 13:**
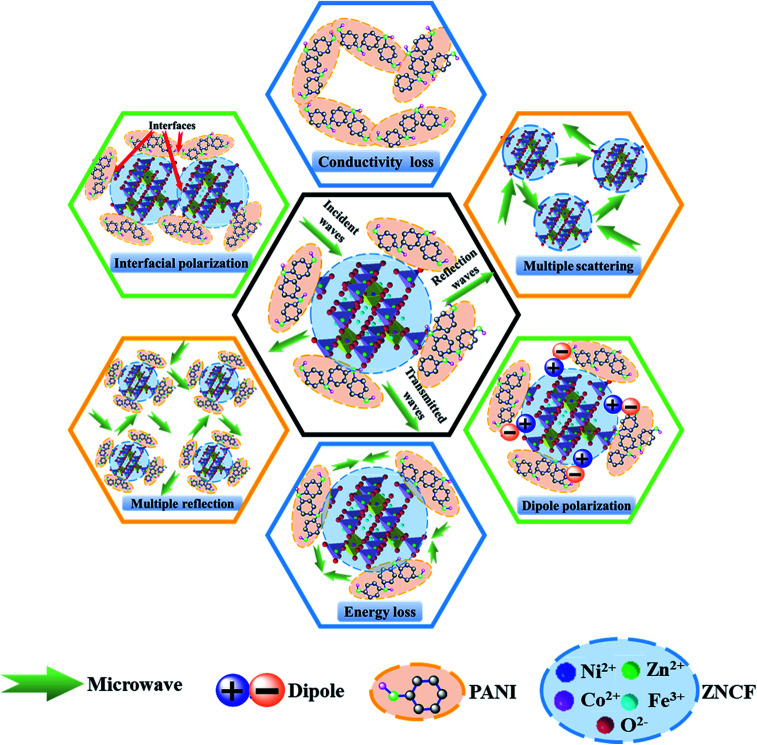
Schematic of microwave absorption mechanisms for the PANI/ZNCF composites.

The excellent microwave absorption properties of PANI/ZNCF composites can be explained as follows (Fig. 13). First, perfect EM wave absorption properties depend on the impedance matching characteristics of composites, which are influenced by permittivity and permeability.^7,8^ If there are clear differences between the values of permittivity and permeability, the EM wave can be reflected from the surface of the composites. In contrast, the EM wave can pass through the surface with slight reflection and then, it enters into the composites with strong absorption.^7,24^ It is well-known that PANI mainly exhibits dielectric loss, whereas ZNCF mainly exhibits magnetic loss, which can be helpful for the impedance matching of their respective composites. Second, because of the large differences of polarity or conductivity between PANI and ZNCF, the electrons or ions in dielectric medium can concentrate at interfaces and exhibit interfacial polarization under the effect of an external electromagnetic field.^12,13^ Moreover, there are a number of gaps and defects in PANI molecular chains, which can introduce dipole polarization.^18,20,25^ The dipole polarization and interfacial polarization can enhance the electromagnetic loss and improve the EM wave absorption. Third, the PANI chains can form a conductive network due to their reasonable conductivity and intensive distribution, which can be beneficial for the transfer of the EM wave.^19^ Finally, when the EM wave passes through the composites, the dense distribution of ZNCF particles and PANI/ZNCF particles can result in multiple scattering and reflection, finally enhancing EM wave absorption. Therefore, the control of PANI chains onto the surface of ZNCF particles is an effective way to enhance microwave absorption applications.

## Conclusion

Novel Zn_0.4_Ni_0.4_Co_0.2_Fe_2_O_4_ grafted with PANI is synthesized *via* the interfacial polymerization method. The XRD and FT-IR results confirm that both NZCF and PANI coexist in PANI/ZNCF composites. The results show that the composites with polymerization at 20 °C for 12 h display excellent EM wave absorption properties; the effective bandwidth (*R*_L_ < −10 dB) is 6.02 GHz, and the maximum *R*_L_ reaches −54.3 dB with a thickness of 6.8 mm. The enhanced microwave absorption properties of PANI/ZNCF composites are mainly related to a special urchin-like structure, which contributes to high dielectric loss and improved impedance matching. These PANI/ZNCF composites with enhanced properties can be efficiently applied in microwave absorption applications.

## Conflicts of interest

There are no conflicts to declare.

## Supplementary Material

RA-008-C8RA05500A-s001
