# Supplementary material for: The effect of polymerization temperature and reaction time on microwave absorption properties of Co-doped ZnNi ferrite/polyaniline composites
Source: RSC Adv. 2018 Aug 17;8(51):29344–55. doi: 10.1039/c8ra05500a (PMC9084490; doi:10.1039/c8ra05500a)
Supplement: RA-008-C8RA05500A-s001 [file RA-008-C8RA05500A-s001.pdf]

Electronic Supplementary Material (ESI) for RSC Advances.
This journal is © The Royal Society of Chemistry 2018

Supplementary Information

The effect of polymerization temperature and reaction time on microwave absorption properties of Co-doped ZnNi ferrite/polyaniline composites

Yiming lei[a,b], Zhengjun Yao[a,b,*], Haiyan Lin[c], Jintang Zhou[a,b,*], Azhar Ali Haidry[a], Peijiang liu[a,b]

Results and discussion

XRD

From the XRD patterns of the obtained PANI/ZNCF composites, shown in Fig. S1a, it is observed that PANI exhibits a weak diffraction peak between 20°-30° owing to the amorphous structure on periodically repeated PANI molecular chains.[17] The XRD patterns of composites show following nine reflection planes (111), (220), (311), (222), (400), (422), (511), (440), and (533), which correspond to 2-theta = 18.7°, 30.8°, 36.1°, 37.7°, 43.9°, 54.5°, 58.1°, 63.7°, and 75.6°. These sharp diffraction peaks indicate the presence of typical spinel cubic structure, which is in good agreement with standard JCPDS Card No. 04-009-3215.[7,18] After the polymerization, all composites exhibit ZNCF diffraction peaks rather than PANI, which means the weak peaks of PANI is concealed by residual carbon and ZNCF as there is no change in crystal structure of ZNCF particles. As shown in Fig. S1b&c, with the increase of reaction time or temperature, the intensities of reflection plane (311) slowly decrease on account of ZNCF particles are coated with more PANI molecular chains which affect the reflection of the crystal surface.[19,20] The average crystallite diameter of ZNCF particle at characteristic peak (311) is 600 nm as estimated by Scherrer's equation.

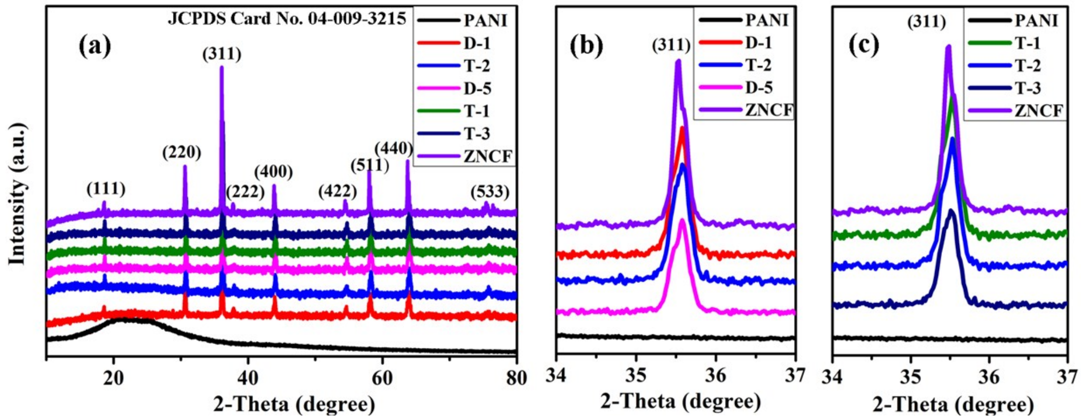

Fig. S1 The XRD patterns of ZNCF, PANI and their composites under different polymerization temperature (T-1, T-2, and T-3) and reaction time (D-1, D-5).
